# Dysphagia in older adults with mild cognitive impairment and dementia through fluoroscopic study with barium swallow in a memory clinic

**DOI:** 10.3389/fneur.2024.1461239

**Published:** 2025-01-15

**Authors:** Georgina Martinez-Peña, Alberto Jose Mimenza-Alvarado, Sara Gloria Aguilar-Navarro

**Affiliations:** National Institute of Medical Sciences and Nutrition Salvador Zubirán, Mexico City, Mexico

**Keywords:** older adults, mild cognitive impairment, dementia, dysphagia, modified barium swallow study

## Abstract

**Introduction:**

Dysphagia and cognitive impairment are prevalent in older individuals. This study aimed to understand the characteristics of dysphagia through fluoroscopy in older adults with mild cognitive impairment (MCI) and dementia.

**Methods:**

A cross-sectional study was conducted at a memory clinic in a tertiary hospital in Mexico City. A total of 158 patients were included, of whom 86 (54.4%) showed a risk of dysphagia, and 84 underwent barium swallow fluoroscopy.

**Results:**

An association was observed between MCI and alteration in the oral phase (OR 0.33, 95% CI 0.12, 0.92, *p* = 0.034). Compared to patients with dementia, patients with MCI showed greater alteration in protection against regurgitation (OR 3.19, 95% CI: 1.05 to 9.72, *p* = 0.042) and in the contraction of the laryngeal muscles (OR 3.54, 95% CI: 1.30 to 9.62, *p* = 0.013).

**Discussion:**

Our findings highlight the altered phases of swallowing in patients with dementia. Additionally, we found a high prevalence of dysphagia in older adults with MCI, underscoring the importance of early detection and intervention.

## Introduction

The aging of the population is a global phenomenon that has significant implications for healthcare. Among the conditions affecting older adults, dysphagia and cognitive impairment are particularly prevalent (1). Patients with dysphagia may exhibit cognitive disorders or language impairments (aphasia) resulting from underlying diseases such as dementia and stroke. Moreover, dysphagia is a geriatric syndrome that affects between 10 and 33% of older adults in general (2). In contrast, the prevalence of dysphagia in patients with dementia can range from 13 to 57% in advanced stages.

Dysphagia occurs due to difficulties in attention, initiative, planning, and execution of daily activities related to the feeding process. These difficulties are reflected in the deterioration of the oral, pharyngeal, and esophageal phases of swallowing and are associated with anatomical and functional alterations in cortical and subcortical regions related to swallowing and feeding (3). It is worth noting that swallowing changes have been identified in patients with Mild Cognitive Impairment (MCI) (4) with an estimated prevalence of 38–63% (5). Even in patients with early Alzheimer’s disease (6) where dysphagia is not clinically evident, it has been demonstrated that oxygen-dependent brain activity is reduced during swallowing compared to healthy controls, suggesting that changes in cortical control of swallowing may begin long before dysphagia becomes apparent (7). In the Mexican population, data are limited. The association between cognition and swallowing disorders suggests the involvement of multiple neuroanatomical systems (8). Dysphagia causes various health problems such as dehydration, malnutrition, and aspiration pneumonia, additionally affecting the patient’s quality of life (9). Many dysphagia patients go undiagnosed and adapt through behavioral changes, while others may experience silent aspiration, it is defined as the entry of food, liquids, or saliva into the airways without the individual showing obvious signs of coughing or discomfort (10).

Some functional Magnetic Resonance Imaging (fMRI) studies have identified the involvement of cortico-subcortical areas in swallowing, particularly related to the activity of the insula, the inferior frontal gyrus, and the anterior cingulate cortex (11). Dysfunctions in these brain areas may contribute to motor impairments during swallowing, leading to difficulties in chewing and bolus formation (11).

The diagnosis and treatment of dysphagia in the early stages of cognitive impairment pose a challenge. From early stages, clinical symptoms of dysphagia may include difficulty initiating swallowing, deviation of the tongue or soft palate, which can lead to problems with bolus formation, cyanosis, unusual respiratory noises after eating, such as wheezing or stridor, persistent coughing after eating, increased gag reflex, delayed swallowing, dry mouth or difficulty forming the bolus, and difficulty coordinating breathing and swallowing (12). There are some clinical assessment scales that attempt to identify symptoms associated with dysphagia. Moreover, diagnostic methods such as video fluoroscopic swallowing studies are considered the gold standard, with a sensitivity of 80% and a specificity of 93%, as they allow the observation of the structural and anatomical stages of swallowing, providing qualitative data on the swallowing motor pattern (11). The objective of this study was to understand the characteristics of dysphagia through fluoroscopy in older adults with MCI and dementia.

## Materials and methods

### Participants

This was a cross-sectional, observational study that included outpatient participants aged 60 and older. All participants underwent clinical and cognitive evaluations at a tertiary care hospital in Mexico City between June 2022 and April 2023. The sample size was calculated based on an estimated dysphagia prevalence of 40%, using the absolute precision method. Sensitivity and specificity were set at 80%, with a precision of 10%. As a result, it was determined that 36 patients per group were needed to test the statistical hypothesis (12).

**Figure 1:**
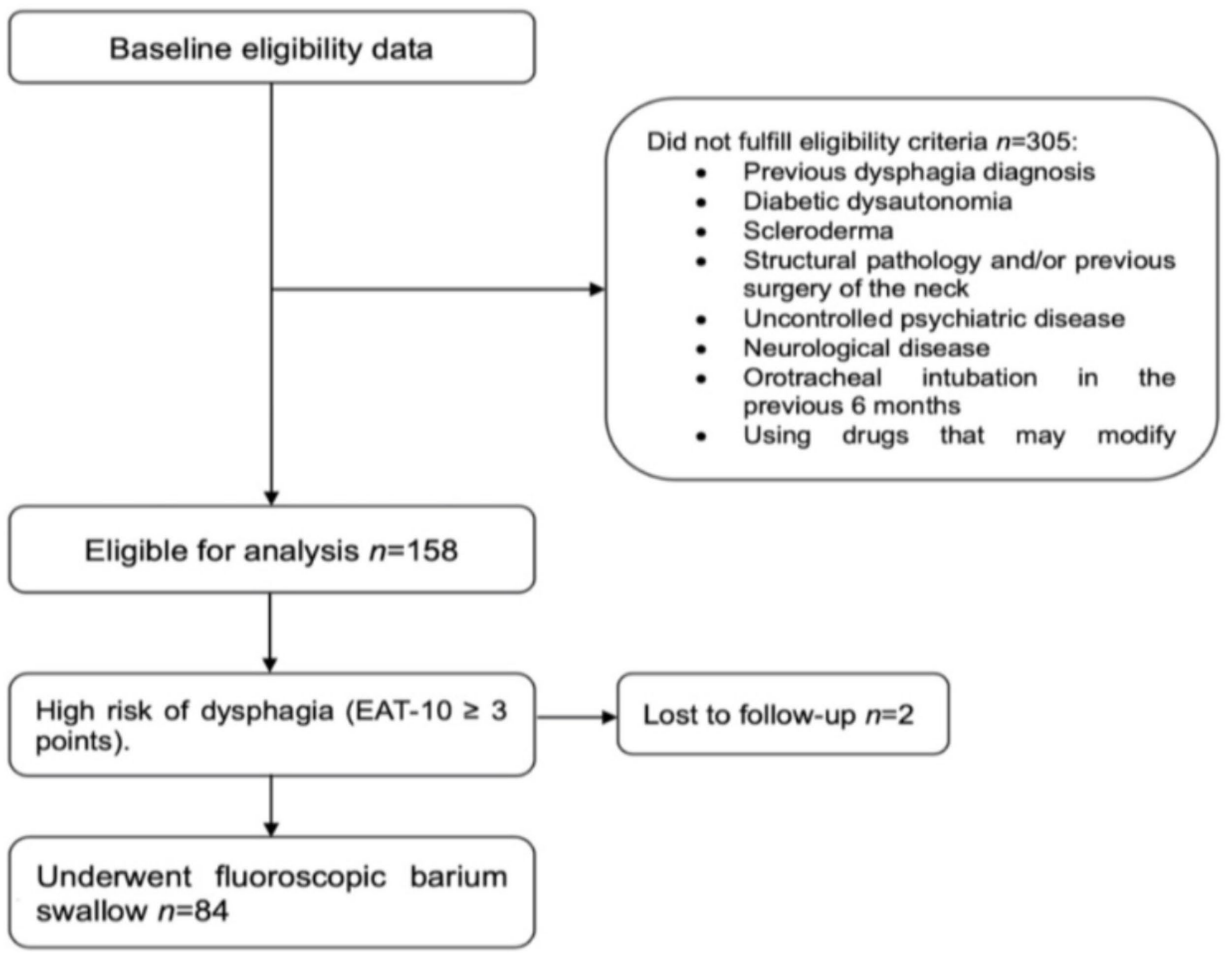
Flow diagram of the participants.

A total of 463 participants with a diagnosis of MCI and dementia were recruited. Of these, 305 were excluded based on specific criteria, which included a previous diagnosis of dysphagia, diabetic dysautonomia, scleroderma, structural pathology, or a history of neck surgery (tumors, polyps). Additional exclusion criteria encompassed uncontrolled psychiatric disorders (depression, anxiety, and/or delirium), neurological diseases (such as Parkinson’s disease, myopathies, myasthenia gravis, and amyotrophic lateral sclerosis), orotracheal intubation in the last 6 months, and the use of medications that could affect swallowing (e.g., benzodiazepines, dopaminergic antagonists, antiepileptics, anticholinergics, antispasmodics, prokinetics, mucolytics, antihistamines, antibiotics, antineoplastics, and anti-inflammatories) (Figure 1).

Finally, 158 participants (66 men, 92 women) were included in the study. The diagnosis and type of MCI were established according to Petersen et al.’s criteria (4), which include memory loss reported by the patient or an informant and a memory score that is 1.5 standard deviations or more below the mean for their age. While cognitive screening tests such as the Mini-Mental State Examination (MMSE) (13) and the Montreal Cognitive Assessment (MoCA) (14) are used to assess general cognition, the standard deviations are calculated from more specific memory tests, such as the California Verbal Learning Test (CVLT), the Wechsler Memory Scale (WMS), and the Rey-Osterrieth Complex Figure Test.

Additionally, participants were required to maintain relatively intact overall cognitive functioning despite specific deficits, which was assessed using an MMSE score of ≥24 or a MoCA score of ≥26, indicating normal global cognitive functioning. Other inclusion criteria included the ability to perform normal activities of daily living and the absence of dementia diagnostic criteria, defined according to the National Institute on Aging and Alzheimer’s Association (NIA-AA) clinical guidance (6). Furthermore, patients had to be able to follow verbal instructions and express a desire to participate in the study. Dysphagia risk was determined using a score of ≥3 on the Eating Assessment Tool-10 (EAT-10) (15, 16).

### Evaluation and assessments

All participants were evaluated by geriatrics and/or neurology specialists. Sociodemographic and health information was obtained from electronic records and categorized based on sex, age, and years of education, as well as the presence or absence of common comorbidities. The functional status for Basic Activities of Daily Living (ADL) was assessed using the KATZ scale, while Instrumental Activities of Daily Living (IADL) were measured with the Lawton and Brody scale. Individuals were considered dependent if they scored less than 5 on the KATZ scale and less than 7 on the Lawton and Brody scale (17, 18). All participants accepted and signed informed consent.

### Modified barium swallow study

The modified barium swallow study was performed on 84 patients at risk of dysphagia according to the EAT-10 (score > 3). A single expert radiologist, who was blinded to clinical evaluation, conducted the assessment and study. The swallowing evaluation included information from the oral (preparatory and transport), pharyngeal, and esophageal phases. Key points assessed included lip closure, bolus retention, bolus propulsion, tongue elevation and posterior displacement, soft palate elevation, upper esophageal sphincter contraction, protection against nasal regurgitation, bolus propulsion by the base of the tongue, epiglottis horizontalization, laryngeal elevation, pharyngeal constrictor muscle contraction, oral and hypopharyngeal cleaning, upper esophageal sphincter relaxation, the bolus entry into the esophagus and return to the initial position of the structures. Additionally, to complete the study, a modified barium swallow study was conducted using three consistencies with contrast medium: solid consistency, prepared by mixing 30 grams of high-density barium sulfate with 5 grams of thickener, diluted with 2.5 mL of drinking water at a temperature of 30 degrees Celsius, and left to rest for 5 min; semisolid consistency, prepared by mixing 30 grams of high-density barium sulfate with 5 grams of thickener diluted with 1 mL of drinking water, left to rest for 5 min; liquid consistency, prepared by mixing 340 grams of high-density barium sulfate diluted with 65 mL of drinking water (19).

With the patient standing or sitting, a lateral and anteroposterior projection was requested, and solid consistency was initially administered in a maximum of 2 mL, with adequate tolerance. If no impairment in the swallowing mechanism was observed in any of the phases, a larger amount of the same consistency was administered. The same steps were repeated for semisolid and liquid consistencies. Each consistency was assessed for the different swallowing phases, and the presence or absence of impairment was defined (20).

### Statistical analysis

A descriptive analysis was conducted to characterize differences between the groups (MCI vs. Dementia). The Chi-square test was used to test differences between categorical variables, and the Mann–Whitney U test was applied for continuous variables. A statistically significant value was considered at *p* < 0.05 for all analyses. Simple and multiple logistic regression analyses were performed to test the probability of having alterations in swallowing phases (dependent variable) based on the presence of cognitive impairment vs. dementia (independent variable with the main effect), adjusting for covariates (age, sex, Lawton scale). Odds ratios (ORs) were obtained from the exponential of the regression coefficients, along with 95% confidence intervals and *p*-values (using the Wald Chi-square test). Statistical analysis was conducted using SPSS software version 25 (SPSS Inc., Chicago, IL, USA).

The protocol was reviewed and approved by the ethics committee and the human research committee of the hospital. Approval code: GER-4207-22-23-1.

## Results

A total of 158 participants were included, of whom 66 (41.8%) were diagnosed with MCI, and 92 patients (58.2%) had dementia (34.8% mild, 17.1% moderate, 6.3% severe). Females constituted 58.2% (*n* = 92) of the sample. The average years of education were 9.2 (5.4), with 39% having 1–6 years of education, 37% having 7–14 years, and 23% having more than 15 years of education. No statistically significant differences were found in terms of comorbidities between the groups. However, 12% of dementia patients had a Body Mass Index (BMI) less than 18.5 kg/m^2^ (*p* = 0.012) (Table 1).

**Table 1:** Socio-demographic and health characteristics.

Characteristics	Cognitive diagnosis		*p* value*
	MCI *n* = 66	Dementia *n* = 92	
Age (years) X̅ (SD)	74.8 (7.9)	81.5 (8.5)	<0.001
>75 years n (%)	31 (48%)	69 (74%)	<0.001
Sexo n (%)			0.174
Female	42 (65%)	50 (54%)	
Dysphagia n (%)	35 (54%)	51 (55%)	0.902
Body Mass Index (BMI) X̅ (SD)	26.5 (4.6%)	23.9 (4.7%)	<0.001
BMI levels n (%)			
<18.5	3 (5%)	11 (12%)	0.012
18.5–24.9	22 (34%)	47 (51%)	
25–29.9	23 (35%)	25 (27%)	
≥30	17 (26%)	10 (11%)	
Social status n (%)			0.701
Single	7 (11%)	7 (8%)	
Married	29 (45%)	47 (51%)	
Widowed	24 (37%)	33 (35%)	
Divorced	4 (6%)	6 (6%)	
Common law marriage	1 (2%)	–	
Education n (%)			0.075
1–6 years	19 (29%)	43 (46%)	
7–14 years	30 (46%)	29 (31%)	
>15 years	16 (25%)	21 (23%)	
MMSE X̅ (SD)	26.3 (2.5)	19.6 (5.8)	<0.001
MoCA X̅ (SD)	20.9 (3.8)	13.3 (5.8)	<0.001
Katz			
Dependent (<5 ADL) n (%)	–	33 (35%)	
Independent n (%)	66 (100%)	60 (65%)	<0.001
Lawton			
Dependent (<7 AIVD) n (%)	16 (25%)	64 (69%)	<0.001
Independent n (%)	49 (75%)	29 (31%)	<0.001
Diabetes mellitus n (%)	25 (38%)	38 (41%)	0.762
Hypertension n (%)	38 (58%)	55 (59%)	0.932
Dyslipidemia n (%)	20 (31%)	29 (31%)	0.956
Atrial fibrillation n (%)	4 (6%)	10 (11%)	0.317

Means and standard deviations for numerical variables. ADL, Basic Activities of Daily Living; IADL, Instrumented Activities of Daily Living. The asterisk (*) next to the *p*-value indicates statistical significance.

Patients at risk of dysphagia based on EAT-10 scores showed 5–16 swallowing abnormalities on the barium swallow fluoroscopy. The distribution of abnormalities included 0.6% with 5 alterations, 1.3% with 6 alterations, 1.3% with 8 alterations, 1.3% with 10 alterations, 2.5% with 11 alterations, 5.7% with 12 alterations, 12.7% with 13 alterations, 19.6% with 14 alterations, 5.7% with 15 alterations, and 2.5% with 16 alterations in the swallowing process.

**Figure 2:**
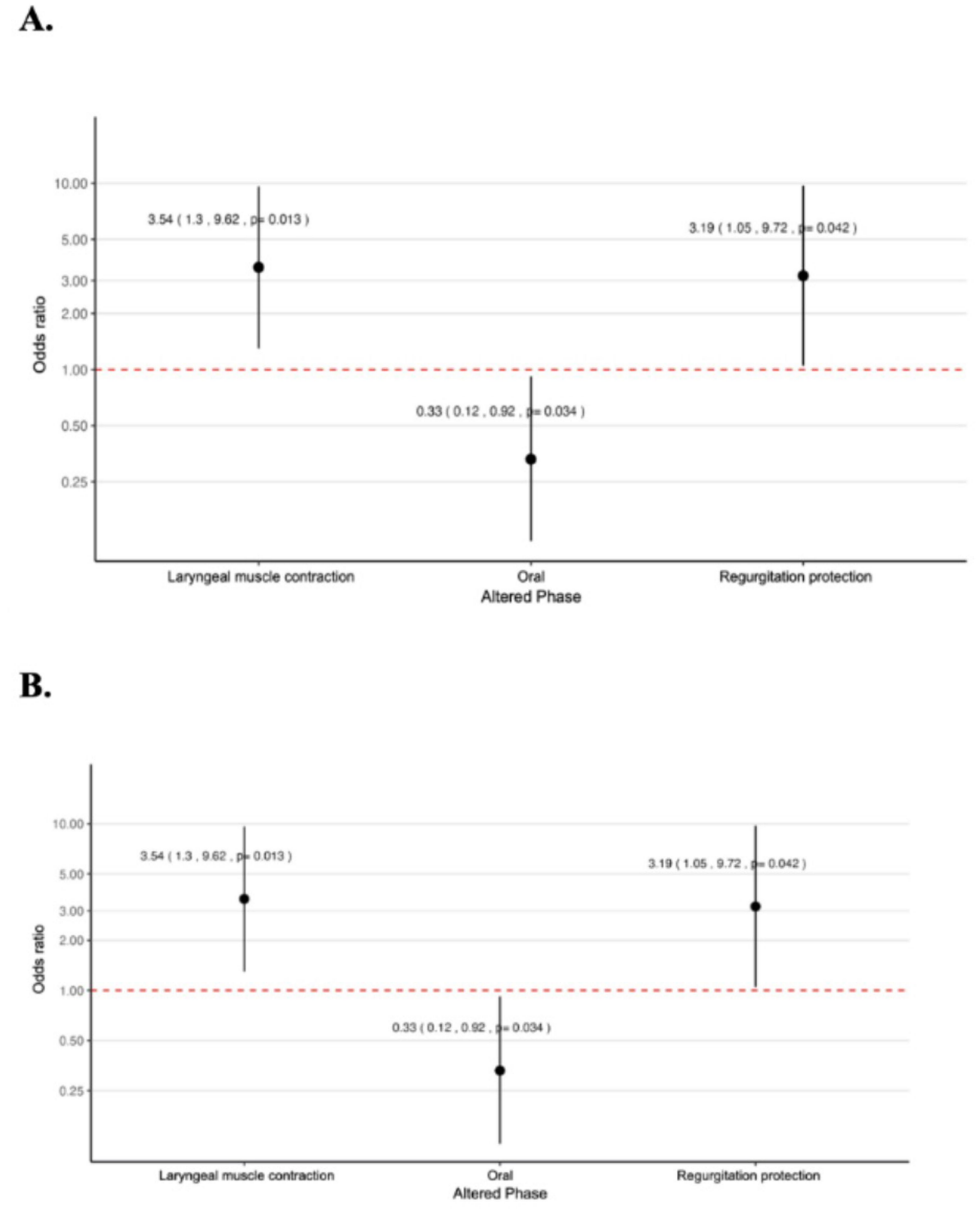
Logistic regression models showing the probability of developing alterations in swallowing phases in mild cognitive impairment versus dementia. OR and CI are shown. **(A)** Simple logistic regression. **(B)** Logistic regression adjusted for age, sex, and Lawton scale score for activities of daily living.

Simple and multiple logistic regression analyses were conducted to analyze the probability of having swallowing alterations (dependent variable) based on the presence of Mild Cognitive Impairment (MCI) versus dementia (independent variable with the main effect). Adjusting for covariates such as age, sex, and Lawton’s functionality scale, the results specifically for the MCI group indicated the following probabilities of altered function: an OR of 0.33 (95% CI 0.12, 0.92) with *p* = 0.034 for alterations in the oral phase, an OR of 3.19 (95% CI 1.05, 9.72) with *p* = 0.042 for protection against regurgitation, and an OR of 3.54 (95% CI 1.30, 9.62) with *p* = 0.013 for contraction of laryngeal muscles (2.54 times associated with the presence of MCI compared to the dementia group). The reciprocal value was 0.22 (Figure 2).

## Discussion

The results of our study demonstrate a high prevalence of dysphagia in older patients with MCI and dementia. These findings are consistent with those reported by Seçil et al., who found that patients with Alzheimer’s disease (AD) in their study had swallowing problems from early stages of the disease. They observed that 75% of patients did not report swallowing complaints; however, they noted that swallowing issues form a continuum, starting from “subclinical dysphagia” to “manifest dysphagia” and ultimately to “swallowing apraxia” (21). Reaffirming this, in some patients, swallowing problems were noted even in the absence of overt complaints.

It appears that eating and swallowing difficulties in people with Alzheimer’s disease (AD) are less severe than in those with frontotemporal lobar degeneration and dementia with Lewy bodies (DLB). Previous studies have looked for the association between eating problems and each of the multiple symptoms, including neuropsychiatric problems in subjects with DLB, and found that the presence of eating behaviors was strongly related to depression and apathy, whereas worsening eating behavior was related to apathy and nocturnal behavior. Importantly, physicians had relatively little knowledge about eating problems in these individuals (22). Our study showed that patients with dementia frequently present dysphagia and, although it was not possible to specify the type of dementia at the time of performing the barium swallow study, our results are consistent with those of previous studies, including the findings of Lages et al. (23). These authors analyzed the relationship between dysphagia and various clinical and cognitive aspects in older adults with dementia. While their study emphasized the influence of external factors, such as place of residence, on dysphagia, the consistency we highlight lies in the association between cognitive impairment and swallowing difficulties. Specifically, Lages et al. observed that dysphagia was more frequent in institutionalized older individuals, which they attributed to a combination of age-related changes and clinical and social aspects of dementia (23).

Another relevant aspect of our study is that patients with dementia presented a low body mass index (BMI), possibly related to dysphagia, which is closely associated with sarcopenia in older adults. The relationship between sarcopenia and dysphagia is bidirectional: sarcopenia can impair feeding capacity and nutrient absorption, potentially leading to food or liquid aspiration into the trachea, resulting in aspiration pneumonia or even death by suffocation in severe cases. In turn, dysphagia limits adequate nutrient intake, contributing to the loss of muscle mass and strength, thus exacerbating sarcopenia. Sarcopenic dysphagia is defined as dysphagia associated with generalized sarcopenia of skeletal muscles (throughout the body) and the muscles involved in swallowing (it is ruled out if there is no sarcopenia in the body) (24).

It is important to consider the effect of sarcopenia as a cause or cofactor of oropharyngeal dysphagia. While retrospective studies, such as one conducted on 109 patients in a nursing home, have not demonstrated a direct association between oropharyngeal dysphagia and sarcopenia, evidence suggests that these factors may be interrelated. The role of sarcopenia as a cause or cofactor needs to be further clarified, particularly to determine whether this condition is an epiphenomenon of severe disease or plays a causal role in the development of oropharyngeal dysphagia. Additionally, prospective studies are needed to explore the specific mechanisms of this bidirectional interaction and provide a better understanding of its clinical impact (25).

In addition, older individuals with dementia, whether mild, moderate, or advanced, often experience dysphagia. This difficulty in swallowing is not only attributable to the dementia itself but also to anorexia and various other associated pathophysiological and behavioral problems (24–26). In our study, a subset of patients underwent barium swallow fluoroscopy. According to the findings, more than half of the patients with mild cognitive impairment had dysphagia, with many swallowing abnormalities. Oszurecki et al. also demonstrated something similar in early stages of the disease (27). As reported by De Stefano et al., patients with MCI, despite having apparent good swallowing or chewing capacity, also presented oral diadochokinesis impairment and food spillage (28). This is primarily due to deteriorated oral motor skills and poor lip function (29). The underlying neural mechanisms are still unclear; however, it has been established that frontal regions are crucial aspects of executive function and eating and swallowing are actions that require cognitive awareness, visual recognition of food, physiological response, planning, and motor execution, as well as structured sensorimotor responses that decrease with the severity of cognitive impairment (31).

Likewise, we also observed a variety of swallowing alterations depending on the stage of cognitive impairment, as the group of patients with MCI had a lower probability of alteration in the oral phase compared to the dementia group. This is a result of swallowing apraxia, lack of coordination in lingual, labial, and mandibular functioning during the oral phase (30). In this phase, the bolus is mixed with saliva, reshaped, and chewed as needed to prepare it for movement to the pharynx. An important finding is that the alteration in protection against regurgitation was 2.19 times more associated with having MCI. On the other hand, we observed that the alteration in laryngeal muscle contraction was 2.54 times associated with the presence of MCI compared to the dementia group.

This finding may be surprising, given that patients with MCI are generally considered to be more neurologically intact than those with dementia. One possible explanation is that compensatory mechanisms within the central nervous system may not be fully effective in preventing laryngeal dysfunction in MCI, even though more generalized cognitive functions may still be preserved. Additionally, early neural damage in MCI could selectively affect the neural pathways involved in laryngeal contraction and airway protection. Although these individuals are in an earlier stage of cognitive decline, specific brain regions responsible for motor control of swallowing may be compromised earlier in the course of the disease.

Another factor to consider is cognitive fatigue, which could disproportionately affect the laryngeal phase of swallowing. Given the complexity of coordinating breathing and swallowing, cognitive fatigue may lead to greater variability or inconsistency in laryngeal muscle contraction and airway protection in MCI patients, even in the absence of other motor deficits. This could explain why the MCI group showed a higher probability of laryngeal dysfunction than the dementia group.

Finally, variability in the progression of neurological damage between individuals with MCI and dementia may account for this finding. While dementia involves widespread cognitive and functional decline, MCI may present with more focal neurological impairments, particularly in areas critical for the fine motor control required for airway protection.

This is a result of the fact that successful swallowing requires a proper connection from the cortex, subcortex, brainstem, and cranial nerves. Initially, it was believed that damage to both hemispheres and the brainstem was necessary for an individual to have dysphagia (5). However, with the advent of neuroimaging techniques, it was demonstrated that dysphagia can result from unilateral lesions of the cerebral cortex (10). Both reflex and voluntary swallowing activate the precentral gyrus, postcentral gyrus, insula, and anterior cingulate gyrus. Additionally, activation has also been observed in the basal ganglia, although their contributions to swallowing are less noticeable than in other mentioned areas (5, 7).

The greater probability of altered laryngeal contraction and protection against regurgitation in MCI highlights the importance of early screening for dysphagia in this population. Clinicians should be aware that patients with MCI, despite their milder cognitive decline, may be at higher risk of developing swallowing-related complications, such as aspiration or pneumonia. Early identification and intervention could be key to preventing serious health outcomes and maintaining quality of life in this group. Furthermore, this finding suggests that dysphagia in MCI may be more complex and nuanced than previously thought, requiring tailored therapeutic approaches to address both the cognitive and motor aspects of swallowing dysfunction.

The results of our study have important clinical implications. Early identification of dysphagia in older individuals with MCI and dementia is crucial to implement appropriate interventions, such as swallowing therapy and dietary modifications, which could improve quality of life and prevent complications related to swallowing and nutritional status, such as aspiration pneumonia. Furthermore, we demonstrated that patients present dysphagia from early stages (MCI); therefore, dysphagia should be systematically sought through clinical or imaging studies, which undoubtedly could contribute to improving comprehensive patient care and their quality of life. We acknowledge that the use of different tests to establish a diagnosis of MCI is a limitation of our study. We also recognize that not having the specific subtype of dementia and/or a control group for each patient in this study is another limitation that can be addressed in future studies.

## Conclusion

Our findings underscore the high prevalence of dysphagia in older individuals with MCI and dementia, as well as its association with greater cognitive impairment. The evidence suggests that patients may experience dysphagia from the early stages of cognitive decline. These results highlight the need to implement effective strategies for the detection and management of dysphagia in the geriatric population to optimize their quality of life and overall well-being.

## Data Availability

The data used in this study are available upon request. Please contact the corresponding author SA-N for access to the data.
